# The evolving role of cardiac magnetic resonance in primary mitral regurgitation: ready for prime time?

**DOI:** 10.1093/ehjci/jey147

**Published:** 2018-10-25

**Authors:** Boyang Liu, Nicola C Edwards, Dudley Pennell, Richard P Steeds

**Affiliations:** 1 Department of Cardiology, University Hospital Birmingham and Institute of Cardiovascular Science, University of Birmingham, Edgbaston, Birmingham, UK; 2 CMR Unit, Royal Brompton Hospital, Sydney Street, London, UK

**Keywords:** cardiac magnetic resonance, primary mitral regurgitation, echocardiography, ventricular remodelling

## Abstract

A fifth of patients with primary degenerative mitral regurgitation continue to present with *de novo* ventricular dysfunction following surgery and higher rates of heart failure, morbidity, and mortality. This raises questions as to why the left ventricle (LV) might fail to recover and has led to support for better LV characterization; cardiac magnetic resonance (CMR) may play a role in this regard, pending further research and outcome data. CMR has widely acknowledged advantages, particularly in repeatability of measurements of volume and ejection fraction, yet recent guidelines relegate its use to cases where there is discordant information or poor-quality imaging from echocardiography because of the lack of data regarding the CMR-based ejection fraction threshold for surgery and CMR-based outcome data. This article reviews the current evidence regarding the role of CMR in an integrated surveillance and surgical timing programme.

## Introduction

Despite clear American Heart Association/American College of Cardiology/European Society of Cardiology (AHA/ACC/ESC) guidelines on the timing of surgery, a fifth of patients with severe primary mitral regurgitation (MR) continue to present post-operatively with reduced ejection fraction and an increased risk of congestive cardiac failure.^1^^,^^2^ These data raise questions regarding the sole use of 2D echocardiography-based dimensions and ejection fraction as Class 1 indications for surgery. Recent guidelines by the American Society of Echocardiography in collaboration with the Society for Cardiovascular Magnetic Resonance acknowledged the role of cardiac magnetic resonance (CMR) but relegated its use to cases where there is discordant information or poor-quality imaging on echo.^3^ CMR has widely acknowledged advantages in monitoring size and function of the LV, and there are preliminary data highlighting discrepancies in quantification of primary MR with echo. This article reviews the current evidence on the role of CMR in an integrated surveillance and surgical timing programme.

## Anatomical assessment of MR: can echo be rivalled?

Complete analysis of the components of the mitral valve (MV) and subvalvar apparatus are required to identify the lesion, aetiology, and type of dysfunction in MR. Such an analysis requires assessment of the leaflets, annulus, chordae tendonnae, and papillary muscles.

### Identification of prolapse and assessment of leaflets

Assessment and measurements on 2D and 3D echo offer high accuracy and reproducibility due to high-temporal resolution and improved frame rates. While 2D transthoracic echo (TTE) is recommended as the first-line examination, 3D TTE acquisitions provide incremental value in establishing the lesion, leaflet, and location of the segments involved, with an accuracy that is equivalent to 2D multiplane transoesophageal echo (TOE).^4^ 3D TOE has the greatest overall accuracy with a higher specificity in the segmental localization of defects (92.8%) compared with surgical findings and is particularly effective in the localization of flail defects (99%). Moreover, 3D TOE has the advantage of producing both static and dynamic measurements of MV anatomy and pathology that can predict both the complexity of repair and likely outcome at surgery, including prolapse height, prolapse volume, and anterior leaflet surface area and leaflet length (Supplementary data online, *Table S1*).^4^^,^^5^

Data comparing echo with CMR for the detection of leaflet abnormalities are limited. CMR requires a dedicated set of sequences that requires time and experience and those most commonly used have lower temporal and spatial resolution than echo.^6^ However, using a set protocol with dedicated valve imaging planes on CMR, one group diagnosed the presence of posterior mitral valve leaflet prolapse with 100% sensitivity and anterior mitral valve leaflet prolapse with 78% sensitivity when compared with 2D TTE, but the study did not compare accuracy to surgical findings.^7^ In another study of 27 patients, CMR correctly identified the presence or absence of a segmental defect in 82% comparing to 2D TTE.^8^ In 43 patients using surgical findings as the reference standard, results on CMR and 2D TOE were concordant in terms of diagnosing prolapse and identification of the leaflet at fault but data on identification of the scallop at fault was not given.^9^ Correct identification not only of the leaflet at fault but of the specific scallop is now the minimum standard in preoperative assessment of primary MR, and the degree of accuracy and amount of information provided by 3D echo far surpasses that of CMR in this respect. Moreover, CMR cannot yet deliver the same high-quality en-face view that is standard in 2D and 3D echo as part of pre-operative planning.

### Annulus

Two difficulties have been encountered in establishing an optimal imaging strategy for the MV annulus: firstly, the surgical reference for sizing is performed in a de-aired heart, and therefore, these measurements do not correspond to ‘live’ imaging; secondly, 3D TOE has highlighted that the annulus is not fixed but dynamic throughout the cardiac cycle, with change in size (early systolic area contraction) and shape (deepening of the saddle-shape). There are no data on the accuracy of CMR-based assessment of MV annular dimensions compared with echo or surgical findings in primary MR, meaning that 3D echo is currently gold standard.^10^

### Assessment of chordae and papillary muscles

The location and attachment of the primary, secondary, and tertiary chordae are critical to current surgical strategies for repair. The location, length, and thickness of the mitral chordae are best measured on 2D echo due to higher spatial resolution and are best visualized on 2D TOE from the transgastric view.^11^ Although the subvalvular apparatus, including chordae, leaflets, and papillary muscle attachments, can be seen on CMR, detailed assessment is much more limited compared with 2D echo which, remains the modality of choice. Assessment of papillary muscle (PM) location, displacement, and function (whether ruptured, fibrotic, infarcted, or displaced) is important for establishing the mechanism of the MR yet there are no comparative data on the utility of echo and CMR in this regard. Data with echo are more compelling; 2D TTE has demonstrated abnormal superior displacement of PM tips during systole as one mechanism contributing to MV prolapse.^12^ 3D TOE has demonstrated PM displacement secondary to progressive LV dilatation with resultant leaflet tethering, as well as the relationship between PMs and LV wall which may assist surgical planning of MV repair.^13^ Although CMR can accurately locate, measure size, and track PM displacement, as well as identify PM infarction/fibrosis,^14^ the clinical implications of these findings in primary degenerative MR remain unclear.

In summary, echo remains the current gold standard for anatomic and functional assessment of the MV apparatus in primary MR, enabling precise localization of the abnormality and determination of aetiology, as well as being key for planning and guiding intervention. There are a few small studies on the role of CMR in defining mitral apparatus anatomy but restricted spatial and temporal resolution remains a limiting factor.

## Severity of MR: a potential role for CMR?

Echo is the principal imaging modality to diagnose and establish severity of MR but requires integration of multiple qualitative, semi-quantitative, and quantitative parameters.^3^ The echocardiographer must also weigh up the different specificity of each parameter and is encouraged to use quantitative measures to grade MR severity, including pulsed Doppler, volumetric and flow convergence [proximal isovelocity surface area (PISA)] methods (Supplementary data online, *Table S1*). Despite the recommendation to quantify MR, a recent communication from the ACC recognized that inadequate quality of echo may be a barrier to optimal care of almost a quarter of patients with MR and that many decisions regarding severity of MR are still made on echo by visually estimating severity based on colour, with a minority of reports providing regurgitant volumes and fraction.^15^ Even when recommendations are followed, quantification by echo is subject to several errors, including practical issues such as over-estimation of non-holosystolic Doppler profiles and failure to account for regurgitant blood volume on the atrial side of the MV annulus in patients with extensive bileaflet prolapse which is not accountable when using a Simpson’s method for quantifying LV end-systolic volume.^16^ Pulsed Doppler methods to estimate differential LV and aortic stroke volumes are valuable, particularly in the presence of multiple jets, constrained and non-holosystolic jets when PISA and vena contracta (VC) are less reliable. However, limitations include the combination of several parameters that carry intrinsic error and increase as the root sum square so that, even under research conditions, the volume bias and limits of agreement between observers for aortic stroke volume (4.7 ± 29.1 mL) and mitral stroke volume (2.9 ± 19.6 mL) are poor.^17^ Likewise, questions have been raised about the reliability of flow convergence even amongst experts from university institutions.^18^ When grading MR as severe or non-severe, the overall interobserver agreement for PISA measurements was poor: 0.37 [95% confidence interval (CI): 0.16–0.58]. A significant source for this variation is the frame-to-frame and beat-to-beat variation in PISA distance which have been quantified as 15–25% even in expert hands, highlighting the importance of multi-frame averaging [estimated at approximately 35 beats if clinicians require an effective regurgitant orifice area (EROA) with CI of ±10%] if precision is to be achieved.^19^ While this may be impractical in routine clinical practice, any degree of averaging will reduce variation. Additionally, the future validation and adoption of automated averaging EROA^20^ and automated 3D colour flow Doppler quantification systems^21^ may enhance the accuracy and consistency of echocardiographic MR quantification.

**Figure 1:**
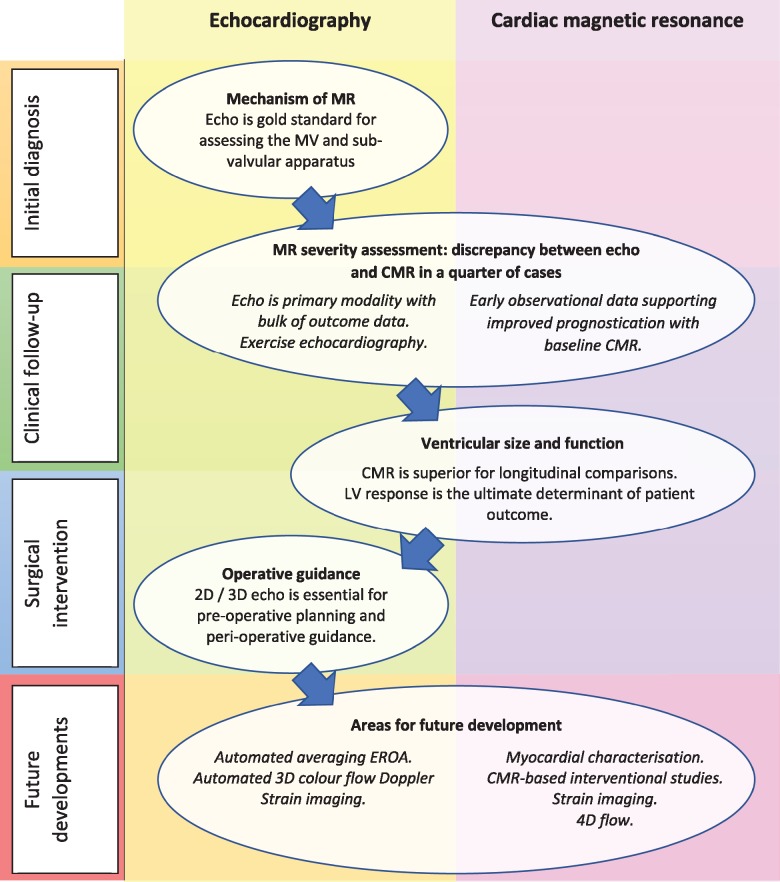
The relative benefits of echocardiography and cardiac magnetic resonance in the management of primary degenerative mitral regurgitation.

In contrast to current guideline-based echocardiographic approaches, estimation of MR severity by CMR (Supplementary data online, *Table S1*) is reliant on a small number of quantitative methods that have been validated against other reference standards, including cardiac catheterization,^22^ quantitative Doppler TTE,^23^ and current single-frame 2D PISA-based methods.^24^ Studies that compare the reproducibility of TTE and CMR in the same patients however are limited. In a prospective study of 26 patients with a range of severity in MR, regurgitant volume was similar between quantitative Doppler, flow convergence TTE, and CMR, but variability in measurements by all methods was poor between observers [Doppler −10 mL (95% CI −76 to 56 mL); PISA: −4 mL (−21 to 13 mL); and CMR 0.7 mL (−30 to 32 mL)].^24^ Interobserver variability of regurgitant fraction was significantly lower by CMR than TTE in a retrospective study of 70 patients (0.1 ± 7.3% vs. −5.5 ± 15%) although this finding only applied to re-analysis of acquired datasets.^25^ Another retrospective study comparing quantitative assessment of MR highlighted that quantification by echo was often not achievable in practice, being feasible only in 44 out of 72 patients.^26^ Where an integrative approach to echocardiographic-based MR severity grading has been applied as per American Society of Echocardiography guidance, intra- and interobserver disagreement is quoted at 20% and 40% on echo.^27^ On CMR based categorization (severe MR defined as regurgitant volume ≥60 mL), discordance is reported at 5% and 10%, respectively. Furthermore, in this prospective observational study of 258 asymptomatic patients, a quarter of patients had a discrepant degree of severity diagnosed on CMR and echo, with the CMR classification offering superior prognostic value for predicting the composite endpoint of mortality or MV surgery during the median follow-up of 5 years.^27^ However, there are also limitations to quantifying MR severity by CMR, including the presence of non-compensated Eddy current-induced fields, variability in volumetric analysis according to placement of the basal slice and in phase velocity mapping according to the plane used for measuring aortic flow.^28^ 4D flow phase contrast CMR may be able to overcome some of these limitations but this currently remains a research tool.^29^ Additionally, echo can easily be combined with exercise testing for cases where exertional symptoms are disproportionate to resting haemodynamics,^30^ whereas this is understandably more challenging for CMR. In summary (*Figure 1*), CMR quantification of MR is simple, feasible in a greater proportion of patients and from the limited comparative studies, may be more reproducible. However, outcome data remain limited and present guidelines are derived from large echo-based outcome studies such that more data are needed which compare the primary analysis of newly acquired CMR datasets.

### Are severity cut-offs equivalent on echo and CMR?

Several studies have found a discrepancy in the grading of severity between CMR and TTE, with a tendency for echo to over-estimate primary MR (*Table 1*).^26^^,^^27^^,^^31^ These data support a role for CMR in such cases where echo grading is qualitative, indeterminate, or incomplete (see *Table 2*). In recent small prospective studies, CMR quantification of regurgitant volume better predicted LV remodelling after surgery [change in left ventricular end-diastolic volume (LVEDV), *r* = 0.85; *P* < 0.0001]; whereas no correlation was present for echo (*r* = 0.32; *P* = 0.1).^31^ In addition, a post-operative decrease in LV internal dimensions most closely correlated (*r* = 0.69) with the regurgitant volume by CMR.^26^ These studies of post-operative outcome both suggested that a lower threshold for severe MR grading may be needed for CMR and is supported by two recent prospective observational studies. In 109 asymptomatic MR patients, severe MR was defined as a regurgitant volume >55 mL or regurgitant fraction >40% using the SVol-AV_f_ method on baseline CMR.^32^ Using these cut-offs, over a mean follow-up of 2.5 years, subjects with a regurgitant volume of >55 mL had a surgery-free survival rate of 21% at 5 years compared with 91% in those with regurgitant volume ≤55 mL. Similarly a second study on 258 asymptomatic patients found a regurgitant volume of ≥50 mL to possess 77% sensitivity and 78% specificity for identifying mortality or indication for MV surgery over a median follow-up of 5 years.^27^

**Table 1:** Example cases highlighting the differing benefits of echo vs. CMR

Case 1: 20-year-old ♀	Case 2: 69-year-old ♀	Case 3: 45-year-old ♂	Case 4: 45-year-old ♂
Moderate on TOE	Severe on TOE	Severe on TOE	MV anatomy
Severe on CMR	Moderate on CMR	Severe on CMR	Echo vs. CMR
Bileaflet myxomatous degeneration resulting in holosystolic CW Doppler, systolic flow reversal in RUPV. MR jet is posteriorly directed, wrapping around LA. VC 6 mm.	Bileaflet myxomatous degeneration resulting in multiple jets. MR is mainly anteriorly directed (VC area 79 mm^2^, systolic flow reversal in RUPV, PISA radius 10 mm, LV SVol − LVOT SVol = 60 mL)	Complex valve lesion with flail A3/P3 scallops from chordal rupture, involving the posteromedial commissure. EROA 70 mm^2^, LV SVol − LVOT SVol = 104 mL, systolic flow reversal in LUPV.	Severe MR due to flail P2/P3 with ruptured secondary chord (arrows) from posteromedial papillary muscle. PISA 14 mm, LV SVol − LVOT SVol = 68 mL, systolic flow reversal in LUPV.
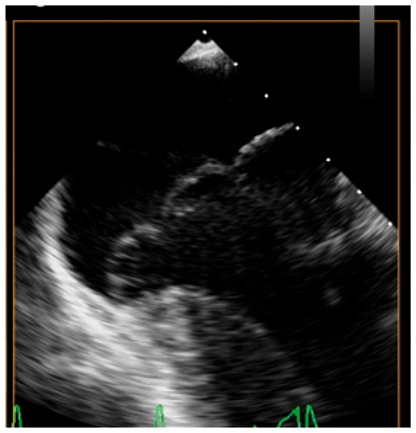	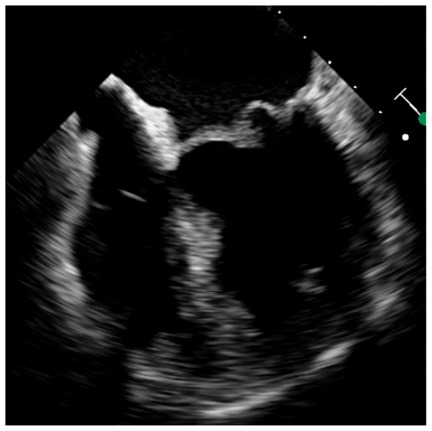	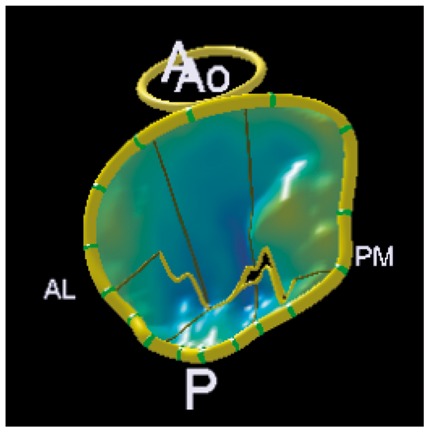	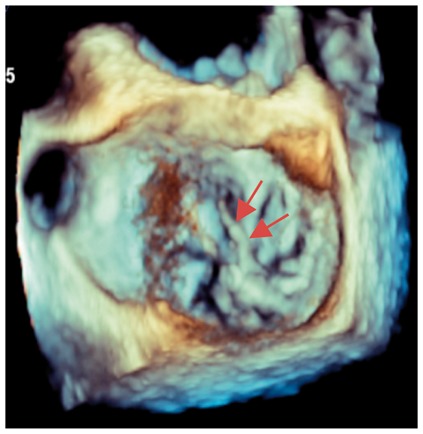
Colour Doppler	Colour Doppler	Colour Doppler	CMR en-face valve view
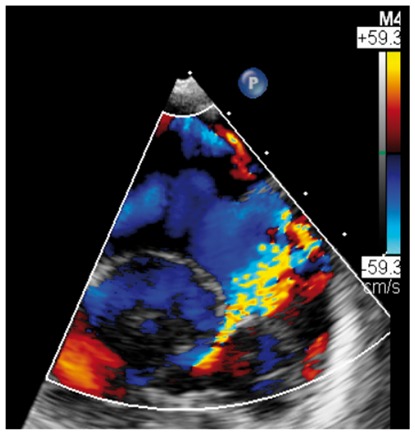	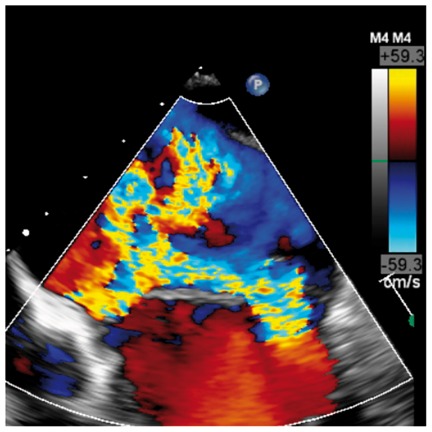	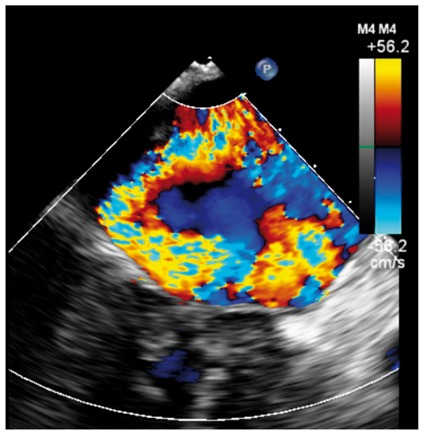	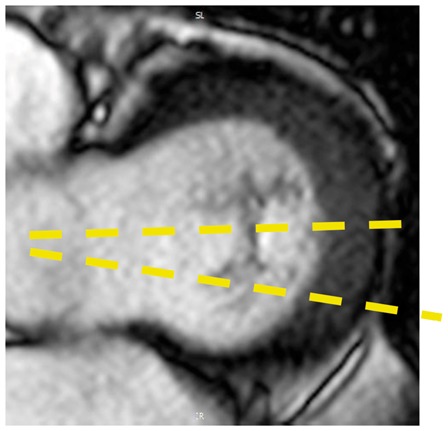
CMR − end systole	CMR − end diastole	CMR − aortic flow	A2P2 and A3P3 scallops
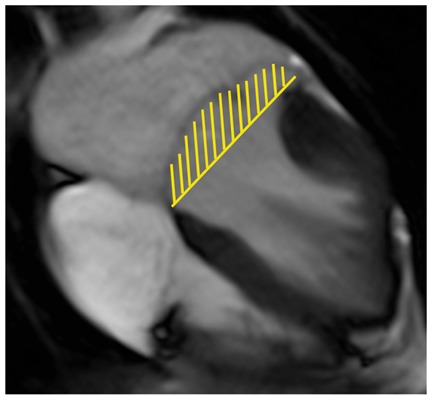	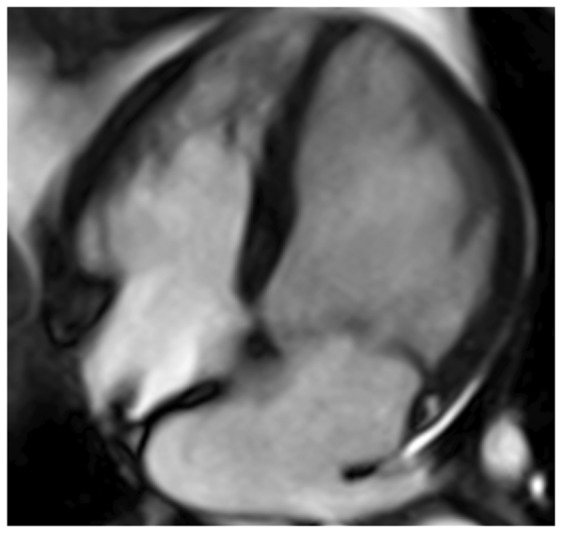	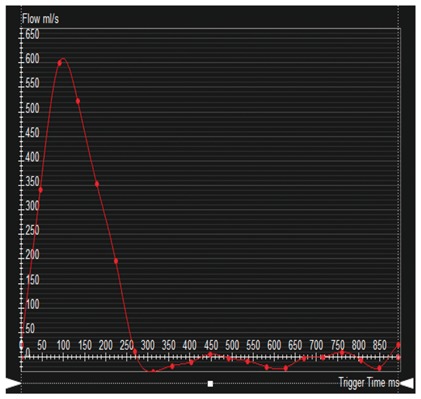	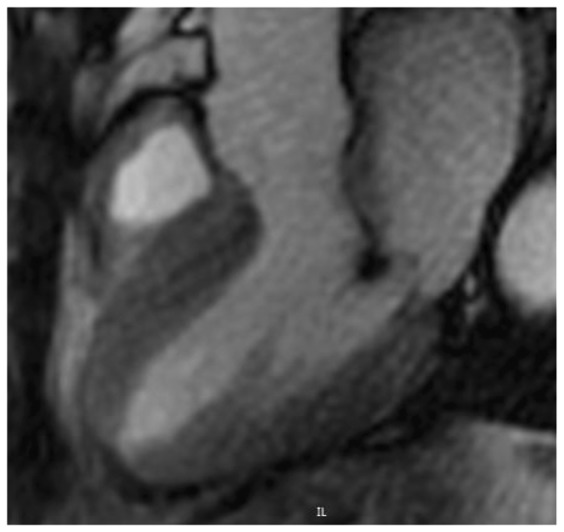
BSA 1.87; LVEDVi 98 mL/m^2^; LVESV 26 mL/m^2^; LVSV 133 mL; LVEF 73%; AVflow 80 mL; LA volume 43 mL/m^2^; MRvol 55 mL; MRfraction 41%.	BSA 1.77; LVEDVi 76 mL/m^2^ LVESVi 18 mL/m^2^; LV SV 102 mL; LVEF 76%; AVflow 72 mL; MRvol 30 mL; MRfraction 30%.	BSA 2.5; LVEDVi 88 mL/m^2^; LVESVi 21 mL/m^2^; LVSV 169 mL; LVEF 76%; MRvol 77 mL; MRfraction 46%.	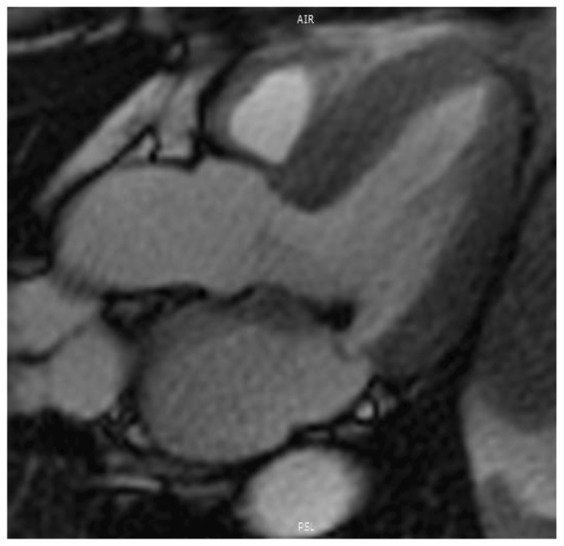

Discussion points: Case 1: VC of 6 mm suggests moderate MR with CMR quantification demonstrating borderline severe MR. Note that the additional end-systolic LV volume below leaflets and above MV annulus (shaded area) in extensive prolapses may not be accounted on Simpson’s biplane, but can be taken into account on a CMR short-axis stack. Case 2: Multiple jets of severe MR visualized on TOE, but repeated longitudinal CMR measuring only moderate MR. Case 3: Agreement between echo and CMR despite the presence of complex MR with commissure involvement. Case 4: Echo remains gold standard for MV anatomy assessment. Scallops can be visualized on CMR using dedicated valve planes, but there is neither 3D visualization nor sufficient spatial resolution for chordal characterization. AVflow, aortic valve flow; BSA, body surface area; CW, continuous wave; LUPV, left upper pulmonary vein; LVEDVi, left ventricular end-diastolic volume index; LVESVi, left ventricular end-systolic volume index; MRfraction, mitral regurgitant fraction; MRvol, mitral regurgitation volume; RUPV, right upper pulmonary vein; SVol, stroke volume.

**Table 2:** Current guideline indications for CMR and TTE and proposed additive value of CMR to these recommendations

Current recommendations for CMR	
Assessment by echo is unsatisfactory Discrepancy between MR severity and clinical findings	ASE/SCMR 2017 ESC/EACTS 2012 AHA/ACC 2014
Current recommendations for TTE	
Moderate MR and preserved LV function requires echocardiographic assessment every 1–2 years Severe MR and preserved LV function requires echocardiographic assessment every 6–12 months	ESC/EACTS 2012 AHA/ACC 2014
Revised recommendations for CMR	
Unsatisfactory assessment by echo, specifically: Indeterminant ‘moderate-severe’ MR. Inability to perform quantitative measurement of MR by echo when severe MR cannot be definitively included or excluded. Borderline ventricular function (LVEF 55–65%) in moderate–severe MR. Fall in LVEF below 60% in asymptomatic patients, where a change of <10% has occurred compared to the previous echocardiographic study. Discrepancy between clinical, exercise, and echo findings (including stress): Symptoms with moderate MR and preserved LV function. Moderate MR with impaired or dilated LV. Routine use of CMR: Moderate MR patients as baseline reference for LV size and function. Every 12–24 months for severe MR with preserved LV function (interdigitating with TTE to give imaging assessment every 6–12 months by one modality).	
Future of CMR imaging in MR?	
Markers of adverse LV remodelling: Strain imaging with tagging or tissue tracking. Fibrosis imaging with LGE and T1/ECV mapping. 4D flow phase-contrast CMR	

ECV, extra-cellular volume; LGE, late gadolinium enhancement.

In summary, single measures of MR severity by echo have wide limits of agreement therefore a multi-parametric approach must be used. While CMR has limitations, it uses fewer parameters and observational data suggest it is better at predicting severity, need for surgery and LV outcomes, although standardized CMR cut-off points for severe MR need to be agreed upon. Importantly, it remains to be seen whether surgical intervention based on CMR parameters alone can lead to an improvement in patient outcome compared with echo.

## Chamber size and function: Achilles heel of echo?

Serial TTE is the recommended method for measurement of chamber size and function in primary MR, with CMR only indicated when echo is ‘unsatisfactory’.^3^^,^^33^ Using a left ventricular end-systolic linear dimension (LVESd) >40 mm on echo is however inherently ‘unsatisfactory’ as a guide to intervention, as it is a poor marker of end-systolic volume in primary MR due to the preferential spherical remodelling process that takes place at the apex and mid-ventricular level.^34^ On the other hand, accurate CMR volumes may be sufficient to monitor patients, since an indexed LVEDV of <100 mL/m^2^ from a single baseline CMR conveyed a 90% surgery-free survival rate at 5 years.^32^ Recognising that delaying surgery until the onset of ventricular dysfunction with left ventricular ejection fraction (LVEF) <60% or LVESd >40 mm, may be associated with an outcome penalty,^35^^,^^36^ the recent AHA/ACC focused update have introduced an additional Class IIa indication as progressive deterioration in LVEF or LVESd on serial imaging.^37^ Finally, mitral regurgitant volume:LVEDV ratio, which is best measured on CMR, has been proposed as an useful indicator that can detect the presence of excessive ventricular dilatation for the degree of MR, thereby highlighting the presence of additional myopathic processes such as ischaemia.^38^ Furthermore, reminiscent of animal models of volume overload where cardiac decompensation contributed to further increases in LVEDV in the presence of static volume overload (Supplementary data online, *Table S2*),^39^ it is reasonable to hypothesize that longitudinal regurgitant volume: LVEDV ratio measurements may be able to identify asymptomatic primary MR patients who are in the early transitional stages of decompensation. For now, although it is not known whether serial imaging by CMR would result in improved patient outcomes compared with echo, the acknowledged greater reliability of CMR volumetric measurements argues for more routine use in surveillance.

LVEF is problematic in MR, whether measured by echo or CMR, as it is a measure of chamber function that is affected by changes in loading conditions. In recognition of this inherent weakness in serial LVEF, recent efforts have focused on identifying more sensitive ‘sub-acute’ markers of LV impairment. These include echo based tissue Doppler (TDI) for measurement of myocardial systolic and diastolic velocities (TDI),^40^ 2D speckle tracking echo for measurement of strain, strain rate, and torsion,^41^ and the CMR equivalents that include grid-tagging and feature/tissue tracking. The Holy Grail of all techniques is to measure detect sub-clinical LV dysfunction in primary MR^42^ although all are affected by loading conditions and none are yet included in current valve guidelines.^43^ Therefore, at present, accurate and reliable measurement of volumes and ejection fraction remain at the centre of management of patients with asymptomatic primary MR and highlights an important area where CMR can be complementary to existing management pathways.

### The left atrium

Left atrium (LA) volume above 60 mL/m^2^ on echo has become a Class IIA indication for MV surgery in the European guidelines, although 2D TTE appears to under-estimate size compared with a 3D CMR approach.^44^ A multi-slice short-axis volumetric approach on CMR is considered the gold standard for structural assessment of the LA (Supplementary data online, *Table S3*) with more accurate and reliable measurement again arguing for a greater role.

### Pulmonary hypertension and the right heart

The onset of pulmonary hypertension represents an adverse complication of MR and a resting peak pulmonary artery systolic pressure (PASP) >50 mmHg on echo is a Class IIA indication to repair.^1^^,^^2^ Although questions have been raised about the accuracy of PASP by echo^45^ and the general estimation of the probability of pulmonary hypertension has not been tested in primary MR,^46^ CMR methods of measuring pressure are not in routine use.^47^^,^^48^ The main benefit of CMR at present lies in assessment of RV chamber size, function, and hypertrophy.^49^ Doppler measurement of TR V_max_ remains central to the management of primary MR and the role of CMR in quantification of RV size and function is supportive only.

## Current limitations and future directions

CMR remains a modality which is less readily available than echo in many centres and which demands expertise. The most commonly used sequences require acquisition over several cardiac cycles and employ breath-holding that some patients cannot manage, although alternatives are now available that cover the entire LV in a fraction of the time and without need for breath-holding.^50^ There is no current direct method to quantify MR severity by CMR and much of the data that compare accuracy of techniques in determining outcomes are derived from small, often single centre studies that do not all demonstrate a consistent advantage using CMR.^51^ However, CMR offers a unique ability to characterize the myocardium and try to unravel the individual myocardial response to chronic MR and help identify those ‘more vulnerable’ to adverse remodelling. Histological evaluation has shown fibrosis on biopsy and CMR techniques of late gadolinium enhancement and T1 mapping allow detection of both irreversible coarse replacement fibrosis and diffuse interstitial fibrosis, independent of loading conditions (Supplementary data online, *Table S3*). In a cross-sectional study of 35 asymptomatic patients with moderate-severe MR and LVEF >60%, diffuse interstitial fibrosis as measured by ECV above 30% was detected in approximately one-third of subjects.^52^ Serial data are needed to determine whether this is associated with decline in LV function or adverse remodelling after surgery and results from on-going prospective studies may help.^53^ The implications of myocardial fibrosis in MR extends beyond ventricular function. There is evidence suggesting that both LGE and diffuse interstitial fibrosis visualized on T1 mapping can predispose to complex ventricular arrhythmias and sudden cardiac death in primary MR.^54^^,^^55^

## Summary: when should you do a CMR for MR?

Any assessment of a patient attending with primary MR must include a detailed echocardiogram using high-quality 2D and 3D imaging to define the aetio-pathogenesis, involving careful assessment of the leaflets, annulus, chords, and papillary muscles. CMR plays a secondary role in this regard. In the assessment of severity of MR, echo may be sufficient in many cases, but CMR can quantify regurgitation in more cases than 2D or 3D and does so with greater reliability. There appears to be a tendency for echo to over-estimate severity of MR and there are data to suggest that CMR measures may deliver better long-term outcomes, although prospective CMR-based interventional studies are lacking. At present, there are sufficient data not only to support the use of CMR where 2D or 3D echo is incomplete, or results are indeterminate but also to promote a greater role in an integrated surveillance and surgical timing programme for patients with moderate-severe MR. CMR has a recognized primacy compared to 2D echo in measurement of LV volumes and ejection fraction, not least due to the limitations of linear measurements on 2D or M-mode in primary MR. This accuracy suggests that CMR should have a bigger part in monitoring, independent of its advantages in measuring atrial and right-sided volumes. In the future, more comparative research is needed to define the roles of each modality against outcomes in prospective studies, and this should also seek to confirm or refute the utility of advanced echo techniques and tissue characterization by CMR.

## Funding

This work was supported by a project grant from the British Heart Foundation [PG/14/74/321056].

**Conflict of interest:** none declared.

## Supplementary Material

Supplementary Tables
