# Supplementary material for: The evolving role of cardiac magnetic resonance in primary mitral regurgitation: ready for prime time?
Source: Eur Heart J Cardiovasc Imaging. 2018 Oct 25;20(2):123–30. doi: 10.1093/ehjci/jey147 (PMC6343082; doi:10.1093/ehjci/jey147)
Supplement: Supplementary Tables [file jey147_supplementary_tables.docx]

**Supplementary table 1.** Direct and indirect methods of MR quantification via echo and CMR. Specific criteria for severe MR are in bold.

|  | | | | **Advantages** | **Limitations** | **Cut off for severe MR** |
| --- | --- | --- | --- | --- | --- | --- |
| **Quantitative quantification** | **Echocardiography** | Stroke volumes method (2D/3D)  (R_Vol_ = SV_MV_ – SV_LVO_)  *Method 1*  R_Vol_ = (Area_MV_ x VTI_MV_) – (Area_LVOT_ x VTI_LVOT_)  *Method 2*  R_Vol_ = (LVEDV – LVESV) - (Area_LVOT_ x VTI_LVOT_) | | Quantitative methods which can be used for multiple or eccentric jets | Different SV_LVO_ can be obtained via (Area_LVOT_ x VTI_LVOT_) or (LVEDV – LVESV)  Measurement errors in annular dimension are squared to calculate area, which is multiplied by error in VTI, resulting in poor inter-observer agreement (1)  Optimal LV volumes and LVOT area require 3D echo +/- contrast | R_Vol_ ≥60ml (2)  R_Fraction_ ≥50% (2) |
|  |  | Flow convergence method by PISA  R_Vol_ = EROA x VTI_reg_  Where:  EROA = 2πr^2^ x Vr / PeakV_Reg_ | | Independent predictive power with outcome (3)  Rapid to perform | PISA inaccurate in presence of eccentric or multiple jets  PISA not applicable when PISA shell is not hemispherical  Multiple measurement errors are compounded  Low expert reproducibility (4) | EROA ≥40mm2: 5-year survival rate of 58±9% on medical therapy; 62±8% risk of a cardiac event (3) |
|  | **CMR** | *Method 1*  R_Vol_ = SV_LV_ – AV_f_ (5)  *Method 2*  R_Vol_ = SV_LV_ – SV_RV_ | | Best correlate of multiparametric grading of MR severity (6) and post-operative reverse LV remodelling (7)  Rvol has direct prognostic implications (8)  During volumetric analysis, RV_SV_ should equate with AV_f_ in the absence of tricuspid regurgitation, acting as an internal quality control | CMR less readily accessible compared to TTE  Increased measurement errors with increasing distance from isocentre of the magnet  Appropriate velocity encoding required  Reliability may be affected by arrhythmia and breath holding | No threshold defined within current guidelines  **R_Vol_ >55ml: surgery-free survival rate of 21% at 5 years (8)**  R_Fraction_ >40%: surgery-free survival of 59% at 5 years (8)  **R_Fraction_ >50%: surgery-free survival of 16% at 5 years (8)** |
| **Qualitative quantification** | **Echocardiography** | | PISA | Fast simple method that assumes a hemispherical convergence of flow towards a circular regurgitant orifice | PISA inaccurate in presence of eccentric or multiple jets  Overestimation when MR not holosystolic | **>1cm at Nyquist 40cm/s** |
|  |  |  | VC width | Easily measured in parasternal LAX view  Good for differentiating mild from severe MR | Limited for multiple jets  Convergence zone needs to be visualised for adequate measurement  Overestimation when MR not holosystolic | **VC ≥0.7cm(2)** |
|  |  |  | 3D VC area on colour Doppler | Direct measurement on 3D echo  Can measure multiple jets of differing directions | Limited temporal and spatial resolution  Subject to colour Doppler blooming  Overestimation when MR not holosystolic  Cumbersome to analyse | 3D VC area >0.4cm^2 (9)^ |
|  |  |  | Colour flow jet area | Easy to measure in apical view | Unreliable, dependent on haemodynamic loading conditions | Colour jet fills >50% of LA or eccentric wall-impinging jet |
|  |  |  | Pulmonary vein flow | Systolic flow reversal in more than one vein is specific for severe MR  Normal pulmonary venous flow suggests absence of severe MR | Mild or moderate MR directed into pulmonary vein can alter flow pattern  Large left atrium may make inaccurate | **Systolic flow reversal** |

2D = 2 dimension, 3D = 3 dimension, Area_MV_ = mitral valve area, Area_LVOT_ = left ventricular outflow tract area, AV_f_ = aortic flow, EROA = effective regurgitant orifice area, LVEDV = left ventricular end diastolic volume, LVESV = left ventricular end systolic volume, PeakV_Reg_ = peak regurgitant velocity, PISA = proximal isovelocity surface area, R_Fraction_ = regurgitant fraction, R_Vol_ = regurgitant volume, SV_LV_ = left ventricular stroke volume, VC = vena contracta, Vr = aliasing velocity, VTI_LVOT_ = left ventricular outflow tract velocity time integral, VTI_MV_ = mitral valve velocity time integral, VTI_reg_ regurgitant flow velocity time integral.

**Supplementary table 2.** Spectrum of adaptive processes in ventricular remodelling with primary mitral regurgitation and corresponding imaging observations.

| **Spectrum of changes in left ventricular remodelling** | **Adaptive processes within the left ventricle** | **Imaging observations** | **Current practice and future directions** |
| --- | --- | --- | --- |
| Initial LV response to volume overload | LV dilatation in volume overload occurs due to increasing metalloproteinase activity and reduced collagen volume fraction, resulting in improved LV compliance (10, 11). In primary MR, this initial LV enlargement increases stroke volume to maintain cardiac output (12). | Initial increase and subsequent decrease in LV compliance on echo based deformation imaging with normal LVEF (13). | Current guidelines recommend regular echo surveillance of asymptomatic severe MR patients (14, 15). |
| “Compensated” chronic mitral regurgitation | Increase in collagen volume fraction and reduction in metalloproteinase activity associated with continued muscle hypertrophy (11, 16).  Variable duration of compensation among different hearts despite no change in burden of volume overload (11). | Expansion of ECV visualised on CMR (17). Subclinical systolic dysfunction as measured by myocardial strain on both echo and CMR thought to be secondary to myocardial fibrosis (18-20).  Potential for myocardial fibrosis quantification to guide timing of surgery (21).  Research required on kinetic energy and LV wall shear stress derived from intracardiac 4D flow, as well as ability for 4D flow to quantify MR in presence of multiple valvular incompetencies and intracardiac shunts (22). | Demand for imaging biomarkers to monitor continued LV remodelling during this “silent” asymptomatic stage, in order to improve the timing of surgery. |
| “Decompensated” chronic mitral regurgitation | Limit of cardiomyocyte hypertrophy reached, with additional rise in metalloproteinase activity resulting in further ventricular dilatation (16). Further ventricular dilatation no longer able to increase stroke volume and worsens myocardial efficiency (12, 23). | CMR gold standard for longitudinal measurement of ventricular volume. “Excess” LV dilatation can be expressed as a RVol/EDV ratio (24).  Overt ventricular failure with symptom onset, LVEF <60% and/or excessive LV dilatation on echocardiography (25-27). | Class I indications for surgery reached, associated with an adverse prognosis (14, 15). |

**Supplementary table 3.** CMR and echocardiographic assessment of chamber size, function and tissue characterisation.

| **Method** | | | **Advantages** | **Limitations** |
| --- | --- | --- | --- | --- |
| **Chamber size** | **Echocardiography** | LV dimension – LVEDd and LVESd measured in PLAX view 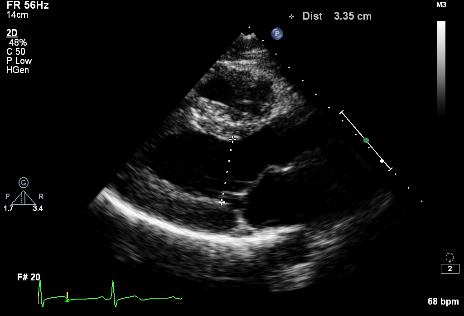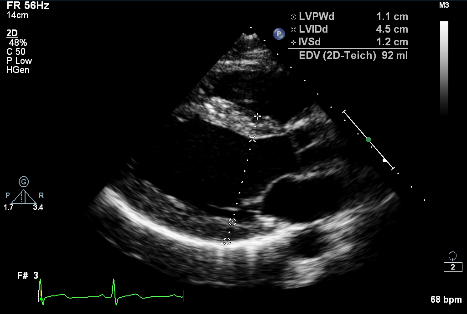 | Easy to acquire  Adopted by current guidelines for surgery | LV dimensions insensitive to the preferential spherical remodelling at the apex and mid-ventricular levels (28) |
|  |  | LV volume - LVEDV and LVESV via Simpson’s Biplane method 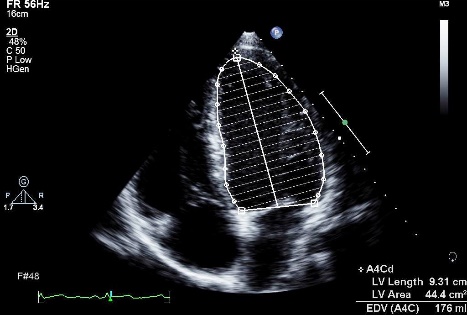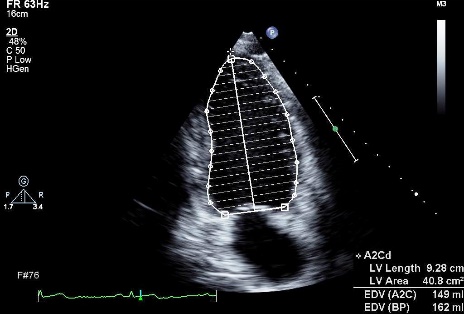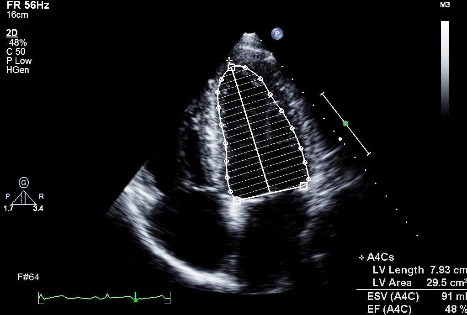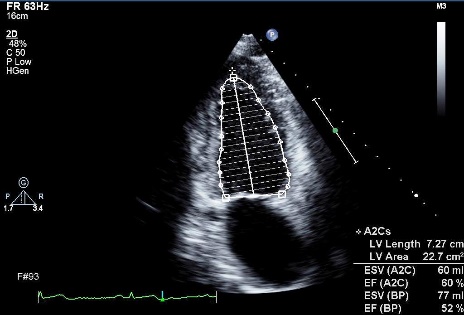 | Easy to acquire  Most widely adopted method | Inadequate endocardial border definition is frequent, particularly in anterior and lateral endocardium |
|  |  | Left atrium – biplane LA volume from 2 and 4 chamber apical views or TOE derived 3D LA volume 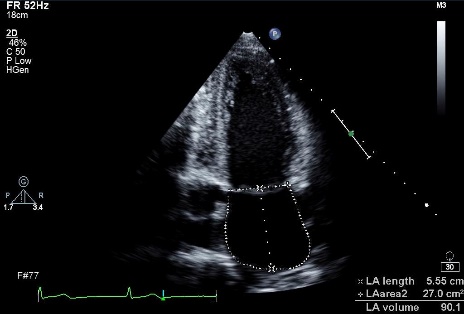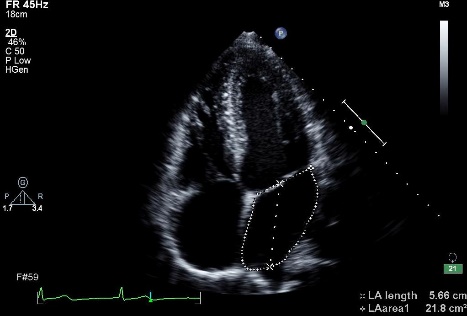 | Easy to acquire  LA volume of ≥60ml/m^2^ is a recognised class II indication for surgery (15) | 2D biplane makes assumptions on LA shape  Both 2D and 3D echocardiographic methods underestimate LA volume compared to 3D CMR (29) |
|  | **CMR** | LV dimension – LVEDd and LVESd measured in 3 chamber LVOT plane 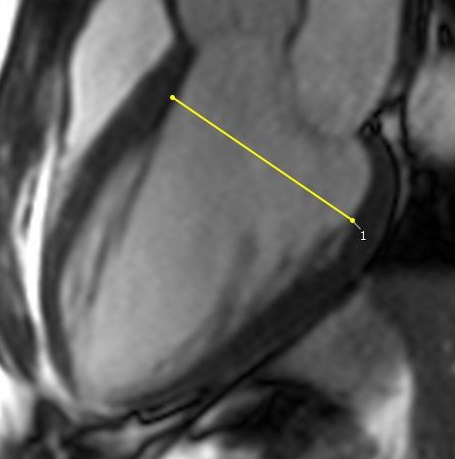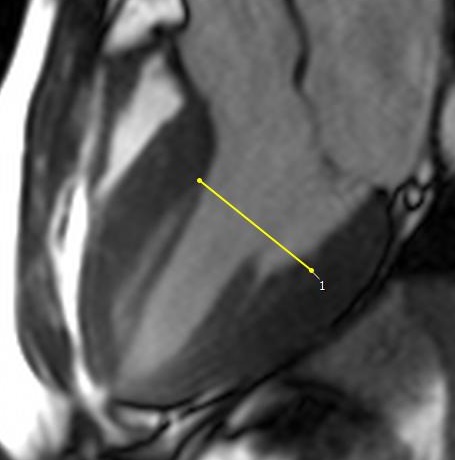 LVESd  LVEDd | Quick to measure | LV dimensions insensitive to the preferential spherical remodelling (arrows) at the apex and mid-ventricular levels (28) |
|  |  | 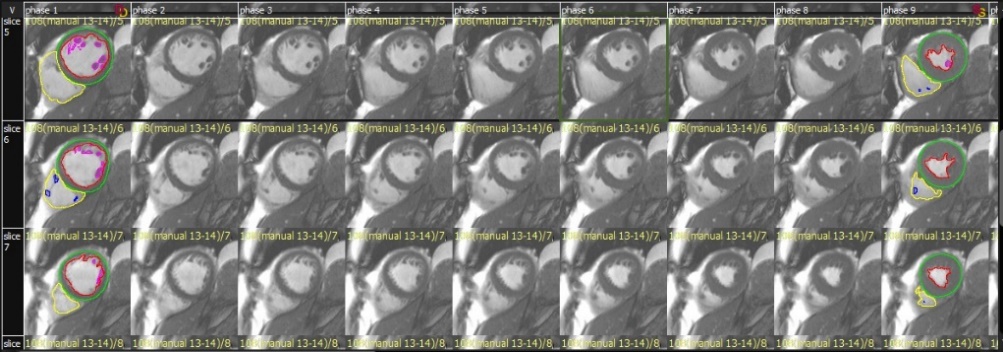LV volume – LVEDV and LVESV from short axis stack | Reference standard for LV volume, interstudy variability of 1±12% (30) | Time consuming contour placement. Different centres adopt different approaches towards the inclusion or exclusion of papillary and trabecular mass from ventricular volume and mass calculations. |
|  |  | Left atrium – best obtained via 3D short axis LA stack 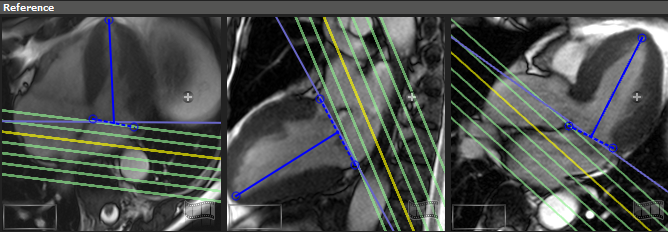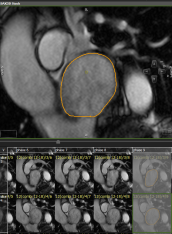 | Reference standard for LA volume measurement - LA enlargement defined as >53ml/m^2^ on CMR (31) | No prognostic data on CMR derived LA volume |
| **Chamber function** | **Echocardiography** | LVEF – diagram as per LV volume  TDI  Speckle tracking 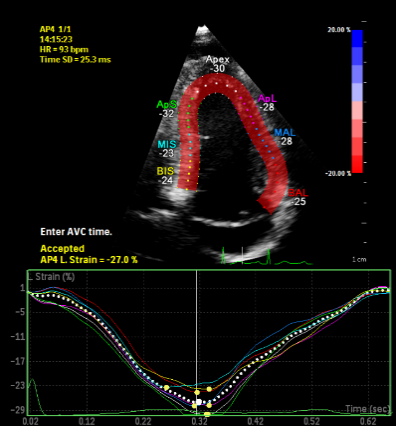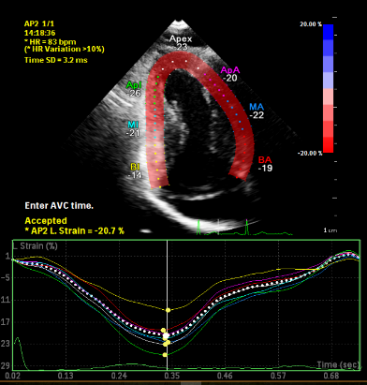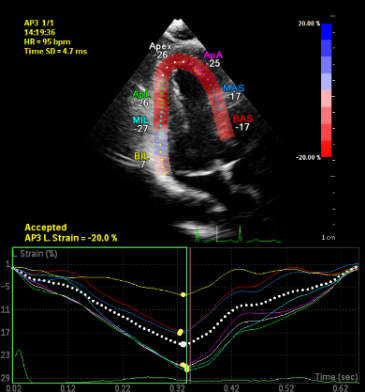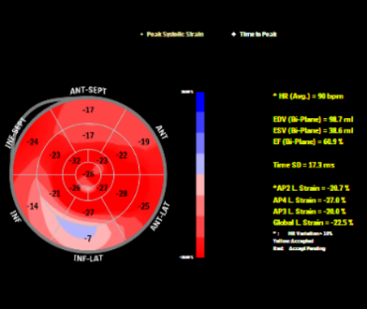 Speckle tracking on 4- 3- and 2-chamber apical views to produce global longitudinal strain | Echo derived GLS is prognostically important in asymptomatic MR with normal LVEF, able to predict postoperative LV dysfunction (32, 33) | LVEF is a poor measure of LV systolic function in MR as it is sensitive to haemodynamic loading conditions  Interstudy variability of LVEF via echo is 6-15% (34) |
|  | **CMR** | LVEF  Tissue tracking 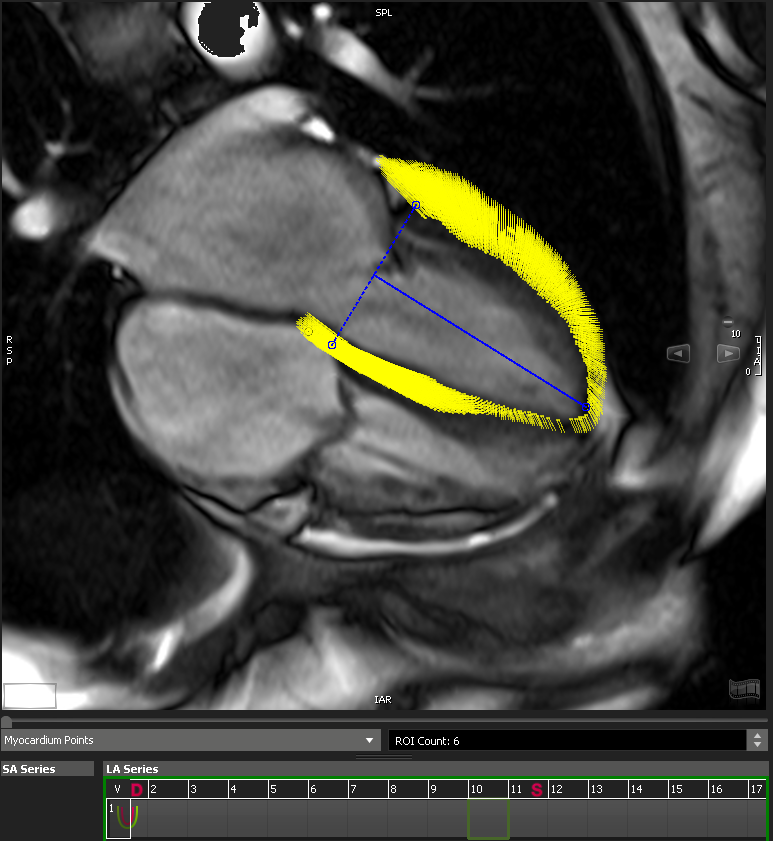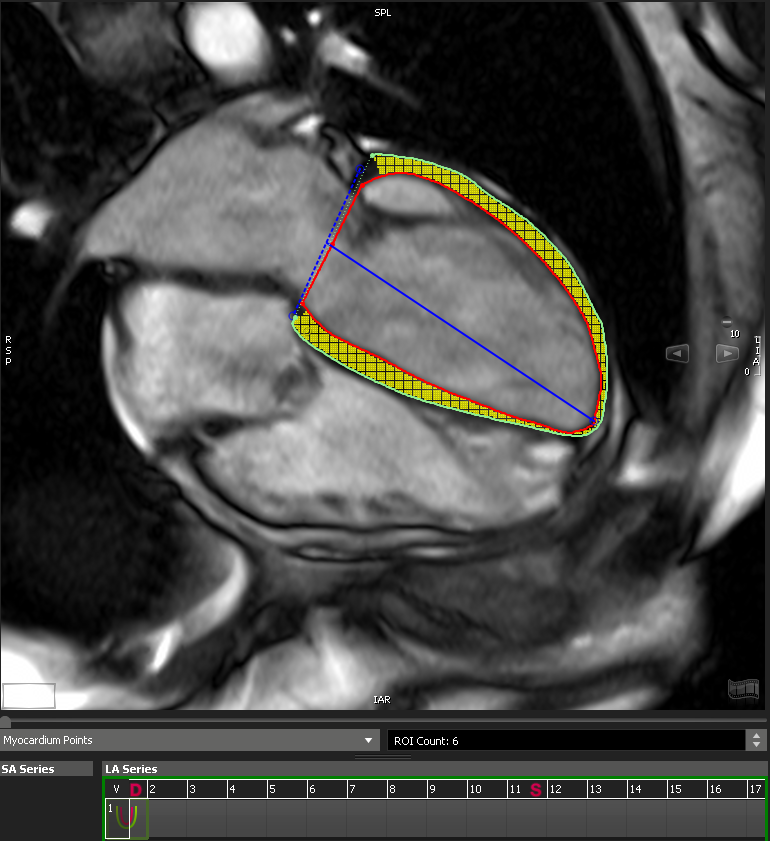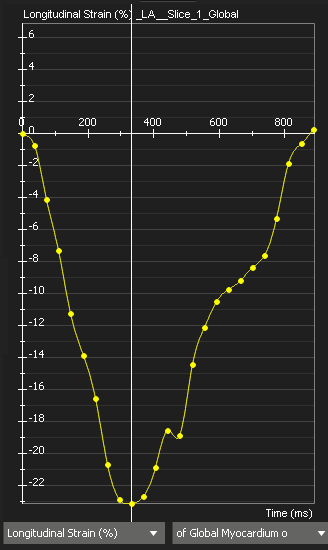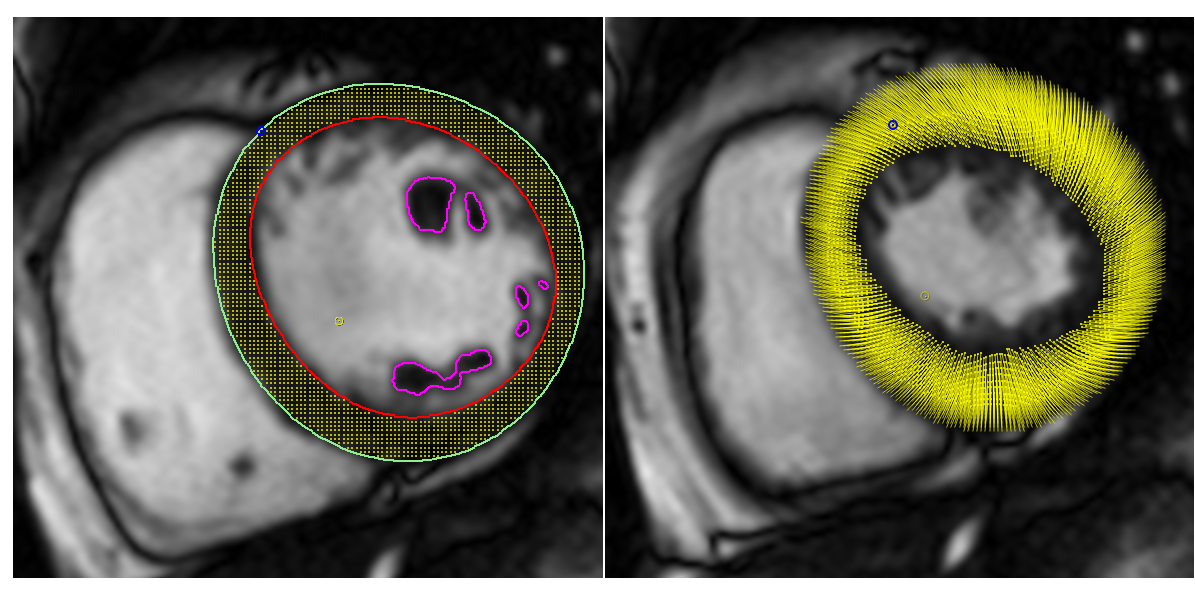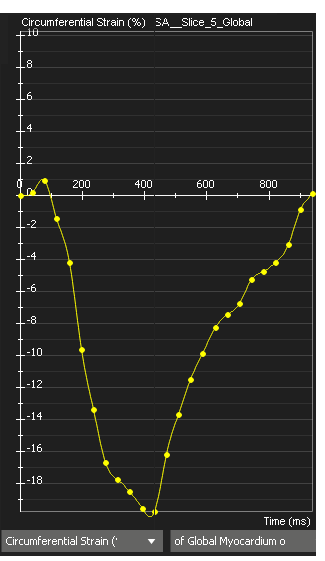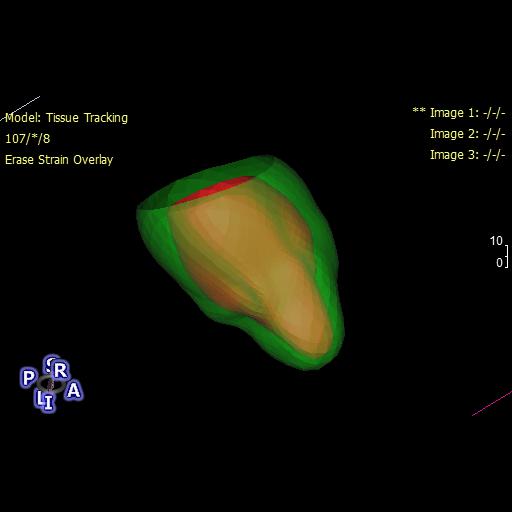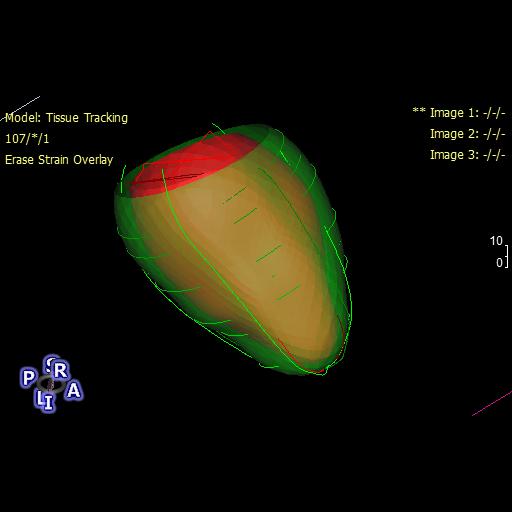 3D tissue tracking on CMR | CMR is the reference standard for measurement of LVEF; interstudy variability of LVEF by CMR is 1±4% (30)  3D tissue tracking reflects the strain pattern of the global myocardium | LVEF limitations are as per echo  No prognostic data available on CMR derived strain / strain rates for primary MR |
| **Tissue characterisation** | **CMR** | LGE  T1 mapping 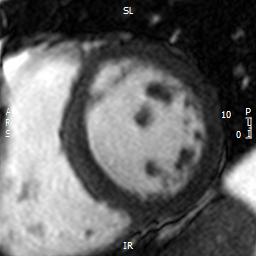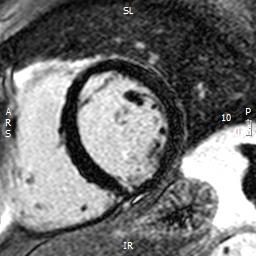 Cine  LGE  T1 mapping 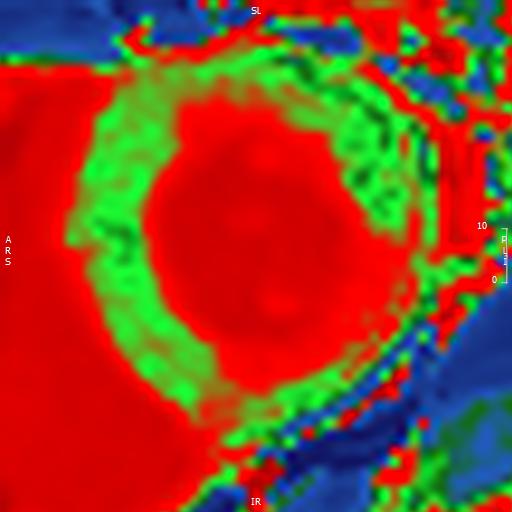  - LGE of the inferomedial papillary muscle and inferior RV insertion point. - LGE representing coarse fibrosis is seen on the T1 map, but additional high T1 region located in the inferolateral LV suggesting the presence of diffuse interstitial fibrosis   T1 map | LGE has been demonstrated to possess prognostic implications across a variety of cardiac pathologies  LGE and T1 may be able to image arrhythmic substrate | There are currently limited outcomes data on LGE and raised T1 times in MR patients |

CMR = cardiac magnetic resonance, LA = left atrial, LGE = late gadolinium enhancement, LV = left ventricle, LVEDd = left ventricular end diastolic dimension, LVEDV = left ventricular end diastolic volume, LVEF = left ventricular ejection fraction, LVESd = left ventricular end systolic dimension, LVESV = left ventricular end systolic volume, LVOT = left ventricular outflow tract, PLAX = parasternal long axis, TDI = tissue doppler imaging.

**References**

1. Thavendiranathan P, Liu S, Datta S, Walls M, Nitinunu A, Van Houten T, et al. Automated Quantification of Mitral Inflow and Aortic Outflow Stroke Volumes by Three-Dimensional Real-Time Volume Color-Flow Doppler Transthoracic Echocardiography: Comparison with Pulsed-Wave Doppler and Cardiac Magnetic Resonance Imaging. Journal of the American Society of Echocardiography.25(1):56-65.

2. Nishimura RA, Otto CM, Bonow RO, Carabello BA, Erwin JP, 3rd, Guyton RA, et al. 2014 AHA/ACC guideline for the management of patients with valvular heart disease: a report of the American College of Cardiology/American Heart Association Task Force on Practice Guidelines. J Am Coll Cardiol. 2014;63(22):e57-185.

3. Enriquez-Sarano M, Avierinos JF, Messika-Zeitoun D, Detaint D, Capps M, Nkomo V, et al. Quantitative determinants of the outcome of asymptomatic mitral regurgitation. N Engl J Med. 2005;352(9):875-83.

4. Biner S, Rafique A, Rafii F, Tolstrup K, Noorani O, Shiota T, et al. Reproducibility of Proximal Isovelocity Surface Area, Vena Contracta, and Regurgitant Jet Area for Assessment of Mitral Regurgitation Severity. Jacc-Cardiovascular Imaging. 2010;3(3):235-43.

5. Chan KMJ, Wage R, Symmonds K, Rahman-Haley S, Mohiaddin RH, Firmin DN, et al. Towards comprehensive assessment of mitral regurgitation using cardiovascular magnetic resonance. Journal of Cardiovascular Magnetic Resonance. 2008;10.

6. Le Goffic C, Toledano M, Ennezat PV, Binda C, Castel AL, Delelis F, et al. Quantitative Evaluation of Mitral Regurgitation Secondary to Mitral Valve Prolapse by Magnetic Resonance Imaging and Echocardiography. Am J Cardiol. 2015;116(9):1405-10.

7. Uretsky S, Gillam L, Lang R, Chaudhry FA, Argulian E, Supariwala A, et al. Discordance Between Echocardiography and MRI in the Assessment of Mitral Regurgitation Severity A Prospective Multicenter Trial. J Am Coll Cardiol. 2015;65(11):1078-88.

8. Myerson SG, d'Arcy J, Christiansen JP, Dobson LE, Mohiaddin R, Francis JM, et al. Determination of Clinical Outcome in Mitral Regurgitation With Cardiovascular Magnetic Resonance Quantification. Circulation. 2016;133(23):2287-96.

9. Zoghbi WA, Adams D, Bonow RO, Enriquez-Sarano M, Foster E, Grayburn PA, et al. Recommendations for Noninvasive Evaluation of Native Valvular Regurgitation: A Report from the American Society of Echocardiography Developed in Collaboration with the Society for Cardiovascular Magnetic Resonance. Journal of the American Society of Echocardiography : official publication of the American Society of Echocardiography. 2017.

10. Brower GL, Chancey AL, Thanigaraj S, Matsubara BB, Janicki JS. Cause and effect relationship between myocardial mast cell number and matrix metalloproteinase activity. American journal of physiology Heart and circulatory physiology. 2002;283(2):H518-25.

11. Brower GL, Henegar JR, Janicki JS. Temporal evaluation of left ventricular remodeling and function in rats with chronic volume overload. American Journal of Physiology-Heart and Circulatory Physiology. 1996;271(5):H2071-H8.

12. Gulch RW, Jacob R. GEOMETRIC AND MUSCLE PHYSIOLOGICAL DETERMINANTS OF CARDIAC STROKE VOLUME AS EVALUATED ON THE BASIS OF MODEL-CALCULATIONS. Basic Research in Cardiology. 1988;83(5):476-85.

13. Borg AN, Harrison JL, Argyle RA, Pearce KA, Beynon R, Ray SG. Left ventricular fitting and diastolic myocardial deformation in chronic primary mitral regurgitation. European Journal of Echocardiography. 2010;11(6):523-9.

14. Nishimura RA, Otto CM, Bonow RO, Carabello BA, Erwin JP, Fleisher LA, et al. 2017 AHA/ACC Focused Update of the 2014 AHA/ACC Guideline for the Management of Patients With Valvular Heart Disease. J Am Coll Cardiol. 2017;70(2):252-89.

15. Baumgartner H, Falk V, Bax JJ, De Bonis M, Hamm C, Holm PJ, et al. 2017 ESC/EACTS Guidelines for the management of valvular heart disease The Task Force for the Management of Valvular Heart Disease of the European Society of Cardiology (ESC) and the European Association for Cardio-Thoracic Surgery (EACTS). Eur Heart J. 2017;38(36):2739-+.

16. Brower GL, Janicki JS. Contribution of ventricular remodeling to pathogenesis of heart failure in rats. American Journal of Physiology-Heart and Circulatory Physiology. 2001;280(2):H674-H83.

17. Edwards NC, Moody WE, Yuan MS, Weale P, Neal D, Townend JN, et al. Quantification of Left Ventricular Interstitial Fibrosis in Asymptomatic Chronic Primary Degenerative Mitral Regurgitation. Circ-Cardiovasc Imaging. 2014;7(6):946-53.

18. Jellis C, Martin J, Narula J, Marwick TH. Assessment of Nonischemic Myocardial Fibrosis. Journal of the American College of Cardiology. 2010;56(2):89-97.

19. Reyhan M, Wang Z, Li M, Kim HJ, Gupta HS, Lloyd SG, et al. Left ventricular twist and shear in patients with primary mitral regurgitation. J Magn Reson Imaging. 2015;42(2):400-6.

20. Witkowski TG, Thomas JD, Debonnaire P, Delgado V, Hoke U, Ewe SH, et al. Global longitudinal strain predicts left ventricular dysfunction after mitral valve repair. Eur Heart J-Cardiovasc Imaging. 2013;14(1):69-76.

21. Liu B, Edwards NC, Neal DAH, Weston C, Nash G, Nikolaidis N, et al. A prospective study examining the role of myocardial Fibrosis in outcome following mitral valve repair IN DEgenerative mitral Regurgitation: rationale and design of the mitral FINDER study. BMC cardiovascular disorders. 2017;17(1):282.

22. Crandon S, Elbaz MSM, Westenberg JJM, van der Geest RJ, Plein S, Garg P. Clinical applications of intra-cardiac four-dimensional flow cardiovascular magnetic resonance: A systematic review. Int J Cardiol. 2017;249:486-93.

23. Linzbach AJ. HEART FAILURE FROM THE POINT OF VIEW OF QUANTITATIVE ANATOMY. Am J Cardiol. 1960;5(3):370-82.

24. Gaasch WH, Meyer TE. Secondary mitral regurgitation (part 1): volumetric quantification and analysis. Heart. 2018;104(8):634-8.

25. Enriquezsarano M, Tajik AJ, Schaff HV, Orszulak TA, Bailey KR, Frye RL. ECHOCARDIOGRAPHIC PREDICTION OF SURVIVAL AFTER SURGICAL-CORRECTION OF ORGANIC MITRAL REGURGITATION. Circulation. 1994;90(2):830-7.

26. Tribouilloy C, Grigioni F, Avierinos JF, Barbieri A, Rusinaru D, Szymanski C, et al. Survival Implication of Left Ventricular End-Systolic Diameter in Mitral Regurgitation Due to Flail Leaflets A Long-Term Follow-Up Multicenter Study. J Am Coll Cardiol. 2009;54(21):1961-8.

27. David TE, Ivanov J, Armstrong S, Rakowski H. Late outcomes of mitral valve repair for floppy valves: Implications for asymptomatic patients. J Thorac Cardiovasc Surg. 2003;125(5):1143-52.

28. Matsumura T, Ohtaki E, Tanaka K, Misu K, Tobaru T, Asano R, et al. Echo cardiographic prediction of left ventricular dysfunction after mitral valve repair for mitral regurgitation as an indicator to decide the optimal timing of repair. J Am Coll Cardiol. 2003;42(3):458-63.

29. Rodevand O, Bjornerheim R, Ljosland M, Maehle J, Smith HJ, Ihlen H. Left atrial volumes assessed by three- and two-dimensional echocardiography compared to MRI estimates. Int J Card Imaging. 1999;15(5):397-410.

30. Thavendiranathan P, Liu SZ, Verhaert D, Calleja A, Nitinunu A, Van Houten T, et al. Feasibility, Accuracy, and Reproducibility of Real-Time Full-Volume 3D Transthoracic Echocardiography to Measure LV Volumes and Systolic Function A Fully Automated Endocardial Contouring Algorithm in Sinus Rhythm and Atrial Fibrillation. Jacc-Cardiovascular Imaging. 2012;5(3):239-51.

31. Maceira AM, Cosin-Sales J, Roughton M, Prasad SK, Pennell DJ. Reference left atrial dimensions and volumes by steady state free precession cardiovascular magnetic resonance. Journal of Cardiovascular Magnetic Resonance. 2010;12.

32. Lancellotti P, Cosyns B, Zacharakis D, Attena E, Van Camp G, Gach O, et al. Importance of Left Ventricular Longitudinal Function and Functional Reserve in Patients With Degenerative Mitral Regurgitation: Assessment by Two-Dimensional Speckle Tracking. J Am Soc Echocardiogr. 2008;21(12):1331-6.

33. Ternacle J, Berry M, Alonso E, Kloeckner M, Couetil JP, Rande JLD, et al. Incremental value of global longitudinal strain for predicting early outcome after cardiac surgery. Eur Heart J-Cardiovasc Imaging. 2013;14(1):77-84.

34. Wood PW, Choy JB, Nanda NC, Becher H. Left Ventricular Ejection Fraction and Volumes: It Depends on the Imaging Method. Echocardiography-a Journal of Cardiovascular Ultrasound and Allied Techniques. 2014;31(1):87-100.
